# Nasopharyngeal Microbiome Signature in COVID-19 Positive Patients: Can We Definitively Get a Role to *Fusobacterium periodonticum*?

**DOI:** 10.3389/fcimb.2021.625581

**Published:** 2021-02-15

**Authors:** Carmela Nardelli, Ivan Gentile, Mario Setaro, Carmela Di Domenico, Biagio Pinchera, Antonio Riccardo Buonomo, Emanuela Zappulo, Riccardo Scotto, Giovanni Luca Scaglione, Giuseppe Castaldo, Ettore Capoluongo

**Affiliations:** ^1^ Department of Molecular Medicine and Medical Biotechnologies, University of Naples Federico II, Napoli, Italy; ^2^ CEINGE Biotecnologie Avanzate S.C.a R.L., Napoli, Italy; ^3^ Task Force on Microbiome Studies, University of Naples Federico II, Napoli, Italy; ^4^ Department of Clinical Medicine and Surgery, University of Naples Federico II, Napoli, Italy

**Keywords:** nasopharyngeal swab, microbiota, next generation sequencing, SARS-CoV-2, *Fusobacterium periodonticum*

## Abstract

Severe acute respiratory syndrome coronavirus 2 (SARS-CoV-2) caused the pandemic Coronavirus Disease 2019 (COVID-19). This virus is highly transmissible among individuals through both droplets and aerosol leading to determine severe pneumonia. Among the various factors that can influence both the onset of disease and the severity of its complications, the microbiome composition has also been investigated. Recent evidence showed the possible relationship between gut, lung, nasopharyngeal, or oral microbiome and COVID-19, but very little is known about it. Therefore, we aimed to verify the relationships between nasopharyngeal microbiome and the development of either COVID-19 or the severity of symptoms. To this purpose, we analyzed, by next generation sequencing, the hypervariable V1-V2-V3 regions of the bacterial 16S rRNA in nasopharyngeal swabs from SARS-CoV-2 infected patients (n=18) and control (CO) individuals (n=12) using Microbiota solution A (Arrow Diagnostics). We found a significant lower abundance of Proteobacteria and Fusobacteria in COVID-19 patients in respect to CO (p=0.003 and p<0.0001, respectively) from the phylum up to the genus (p<0.001). The *Fusobacterium periodonticum* (*FP*) resulted as the most significantly reduced species in COVID-19 patients respect to CO. *FP* is reported as being able to perform the surface sialylation. Noteworthy, some sialic acids residues on the cell surface could work as additional S protein of SARS-CoV-2 receptors. Consequently, SARS-CoV-2 could use sialic acids as receptors to bind to the epithelium of the respiratory tract, promoting its clustering and the disease development. We can therefore speculate that the significant reduction of *FP* in COVID-19 patients could be directly or indirectly linked to the modulation of sialic acid metabolism. Finally, viral or environmental factors capable of interfering with sialic metabolism could determine a fall in the individual protection from SARS-CoV-2. Further studies are necessary to clarify the precise role of *FP* in COVID-19.

## Introduction

The severe acute respiratory syndrome coronavirus 2 (SARS-CoV-2) is the cause of pandemic Coronavirus Disease 2019 (COVID-19) (Zhu et al., 2020). Most COVID-19 patients show an acute respiratory distress syndrome and typical symptoms including fever, dry cough, and tiredness (He et al., 2020). Other patients suffer from pain, nasal congestion, anosmia, sore throat, or gastrointestinal illness such as diarrhea (Khatiwada and Subedi, 2020). Moreover, a large part of subjects resulting positive at the SARS-CoV-2 molecular assay, especially during this current pandemic wave, are asymptomatic carriers of the virus in population (Khatiwada and Subedi, 2020). There are many factors (genetic, comorbidities, age, gender) that can influence both the onset of disease and the relative severity of its complications (Abu Hammad et al., 2020; Russo et al., 2020). Among these, the microbiome composition at different levels (gut, lung, skin) was also investigated. Microbiome, the collective genomes of all microorganisms living in our body, in fact, can play a pivotal role in the development of several diseases (Wang et al., 2017; Nardelli et al., 2020; Sacchetti and Nardelli, 2020; Scaglione et al., 2020). In fact, a perturbation of the microbial composition, named dysbiosis, could decrease the microbiota diversity, changing its composition, and promote an inflammatory environment favoring the coronavirus invasion and viral replication (Antunes et al., 2020). The latter induces a strong inflammatory response with the consequent massive release of cytokines and chemokines (the so-called “cytokine storm”). The latter results in a systemic damage, with multiorgan injury, particularly in patients with severe COVID-19 (Abu Hammad et al., 2020). Recent publications showed the possible relationship between gut, lung, nasopharyngeal, or oral microbiome and COVID-19 (Antunes et al., 2020; Bao et al., 2020; De Maio et al., 2020), but very little is known about it. Therefore, we aimed to verify if the nasopharyngeal microbiome could influence both the development of COVID-19 and the severity of its symptoms. To this purpose, we analyzed the nasopharyngeal microbiome in COVID-19 patients and control individuals in order to investigate the possible association between the microbiome composition and feature of COVID-19.

## Materials and Methods

### Patients and Controls

Thirty-eight subjects were included in the study between May and September 2020. These individuals were divided in three groups: n=12 controls (8 females and 4 males; age range: 30–60) who resulted negative at the SARS-CoV-2 molecular assay; n=18 symptomatic COVID-19-positive patients (6 females and 12 males; age range: 35–84 years; namely T0) who were submitted to nasopharyngeal swab at the admission within the Department of Malattie Infettive, University of Naples Federico II. The severity of the symptoms was evaluated in according to the Clinical Status Ordinal Scale as reported by Beigel et al. (2020).

When possible, a second swab was collected one week after the recovery (T1; n=8 patients). The COVID-19 diagnosis was performed combining the clinical features with the SARS-CoV-2 RNA detection by using real-time reverse transcriptase-polymerase chain reaction (RT-PCR) assay on the nasopharyngeal swabs. This analysis was performed in the COVID-19 molecular reference Lab n. 777777 of CEINGE Biotecnologie Avanzate S.C.a R.L., belonging to the CORONET Campania Regional network for SARS-CoV-2 diagnostics.

The study was approved by the Ethical Committee of the University Federico II of Naples (authorization n.180/20/ES1 on 25.05.2020). All the enrolled subjects signed the informed consent to participate in the study: our research was conducted in accordance with the Helsinki Declaration policy (2013). The clinical and anamnestic data of each subject, collected by the clinicians, are reported in **Table 1**.

**Table 1:** General and biochemical characteristics of n=18 COVID-19 patients.

Parameters	T0	T1
**Age range (years)**	35–84	
**Gender**	6 F/12M	
**Weight (Kg)**	75.5 ± 16.4	
**Height (m)**	1.7 ± 0.03	
**Red blood cells (U/L)**	4306250 ± 854416	3915000 ± 568984
**White blood cells (U/mL)**	7438.75 ± 4557.9	7776.2 ± 3573.6
**Neutrophils (U/mL)**	5675 ± 4105	4481.3 ± 2525.3
**Lymphocytes (U/mL)**	1238.75 ± 652.8	1472.5 ± 512.6
**Hemoglobin (g/mL)**	12.3 ± 2.2	11.0 ± 1.4
**Platelets (U/mL)**	273250 ± 227449	234875 ± 137843
**Aspartate aminotransferase (U/L)**	25.3 ± 12.3	28.25 ± 8.1
**Alanine aminotransferase (U/L)**	15.5 (11.7–31.0)	18.5 (13.2–41.7)
**Creatinine (mg/dL)**	1.2 ± 1.0	1.1 ± 1.0
**Azotemia (mg/dL)**	50.6 ± 34.7	53.4 ± 39.4
**Creatine Phosphokinase (U/L)**	24 (15.7–38.7)	40.5 (28.5–79.7)
pro-BNP* (pg/mL)	74.3 ± 62.8	72.4 ± 51.8
**Prothrombin time (s)**	1.1 ± 0.08	1.1 ± 0.08
**Partial thromboplastin time (s)**	1.0 ± 0.1	1.0 ± 0.1
**D-Dimer (ng/mL)**	2.0 ± 1.6	2.2 ± 1.6
**Fibrinogen (mg/mL)**	476.5 ± 118.2	449.6 ± 134.3
**Reactive C protein (mg/L)**	4.1 (1.4–9.9)	2.0 (0.4–4.3)
**Inteleukin-6 (pg/mL)**	21.1 (9.0–29.8)	9.9 (5.5–20.2)
**Ferritin (ng/mL)**	248.5 (214.2–423.2)	264.5 (131.5–597.7)

Data are represented as mean ± standard deviation or median (25°–75° percentiles) for non-parametric variables.

*N-terminal prohormone of brain natriuretic peptide.

### Sample Collection and Storage

For each individual we collected a dedicated nasopharyngeal swab for the molecular assay using the sterile cotton swabs (COPAN SPA, Brescia, Italy). An aliquot of these swabs was stored at -80°C until used. The swab sample was drawn at T0 before starting any drug treatment.

### DNA Isolation

All swab samples were thawed at room temperature and bacterial DNA was isolated using MagPurix^®^ Bacterial DNA Extraction Kit (Zinexts Life Science, New Taipei City, Taiwan), according to manufacturer instructions. All extractions were performed in a pre-PCR designated room in a COVID laboratory next the CEINGE. DNA samples were stored at −20°C until further processing. The yield and the quality of extracted DNA were determined using Qubit dsDNA HS (High Sensitivity) assay kit (Invitrogen Co., Life Sciences, Carlsbad, USA) run on the TapeStation (Agilent Technologies, Santa Clara, CA, USA).

### 16S rRNA Sequencing and Data Analysis

The hypervariable V1-V2-V3 regions of the bacterial 16S rRNA were amplified using Microbiota solution A (Arrow Diagnostics, Genova, Italy) according to the manufacturer instructions. The quality and quantity of amplification products were evaluated by TapeStation system and Qubit dsDNA BR assay. This step is preliminary to the pooling procedure consisting of equimolar libraries. Sequencing of our libraries was performed on MiSeq Illumina^®^ sequencing platform (Illumina, CA, US) using V2 500 cycles reagent. Sequencing data were analyzed by a dedicated bioinformatics software (MicrobAT Suite - SmartSeq, Novara, Italy) able to decipher the operational taxonomic units (OTUs). Statistical analyses were performed by MicrobiomeAnalyst program (https://www.microbiomeanalyst.ca/) using default parameters. The alpha diversity was evaluated using the Chao-1, Shannon, and Simpson indices, respectively. Wilcoxon rank-sum test (Mann-Whitney) was performed to test the significance of pairwise richness differences. The beta-diversity has been evaluated through non-metric multidimensional scaling (NMDS) ordination of variance stabilised counts of taxa for CO and COVID positive samples, compared using Bray-Curtis dissimilarity. Permutation analysis of variance (PERMANOVA) and corresponding R-squared and p-values were calculated. By Kruskal-Wallis Rank Sum Test, the abundance of taxa, at each taxonomic level, were compared. Comparisons were performed between either CO and COVID+ or CO and COVID+ (at T0 and T1 of observation). The Raw and processed high-throughput sequencing data have been deposited in the Sequence Read Archive (SRA) (https://www.ncbi.nlm.nih.gov/sra) under Project SUB8444038.

## Results

The sequencing of all nasopharyngeal swabs produced an average of 67,500 counts per sample.

**Figure 1:**
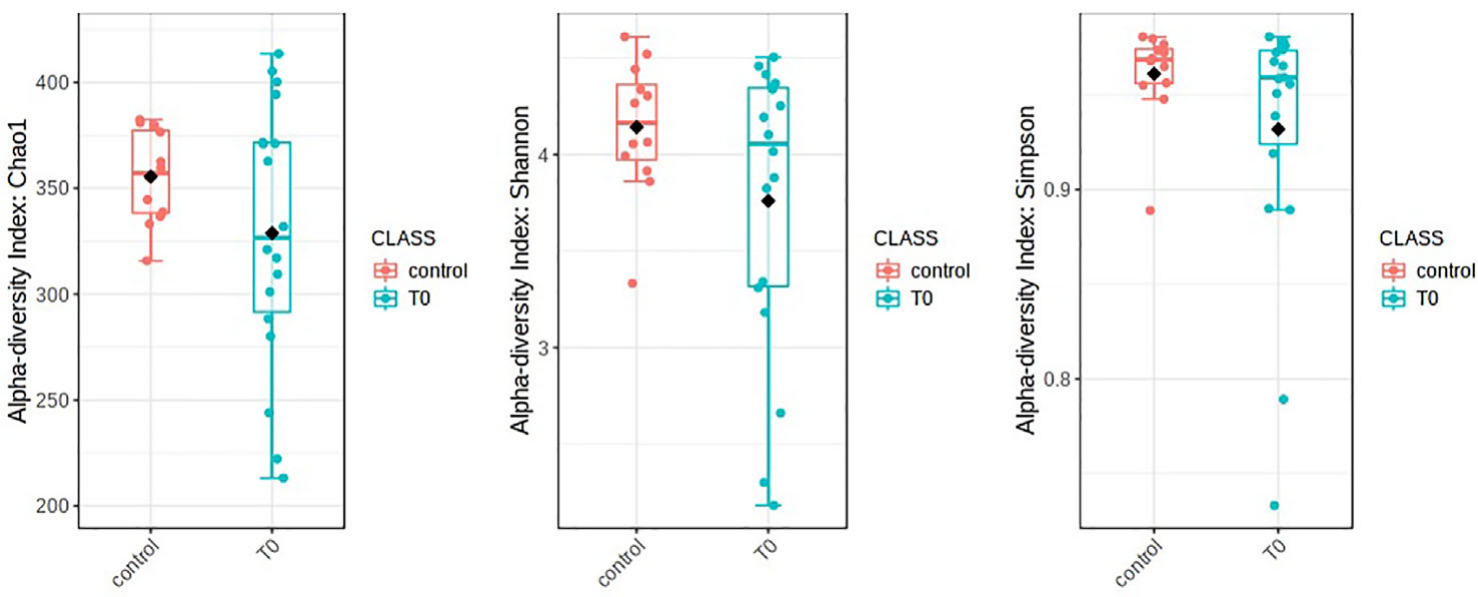
Alpha diversity in patients with COVID-19 (T0) in respect to control subjects. Alpha diversity analysis was evaluated by means of several metrics: Chao1, Shannon, and Simpson indices. Overall, the plots do not show any significant difference between T0 and Control (statistical analysis by Wilcoxon rank-sum test Mann-Whitney). Nevertheless, a soft trend of decreased richness in T0 respect to Control was observed.

The nasopharyngeal microbiome was different in patients with COVID-19 (T0) in respect to control subjects (CO). Alpha diversity was evaluated through Chao1, Shannon, and Simpson diversity indices, the latter measuring the within-sample diversity and comparing species richness between the analyzed groups. All indexes resulted as not statistically different for the comparisons between COVID-19 patients and CO (Chao1: p=0.28, Shannon: p=0.27, and Simpson: p=0.32) (**Figure 1**).

**Figure 2:**
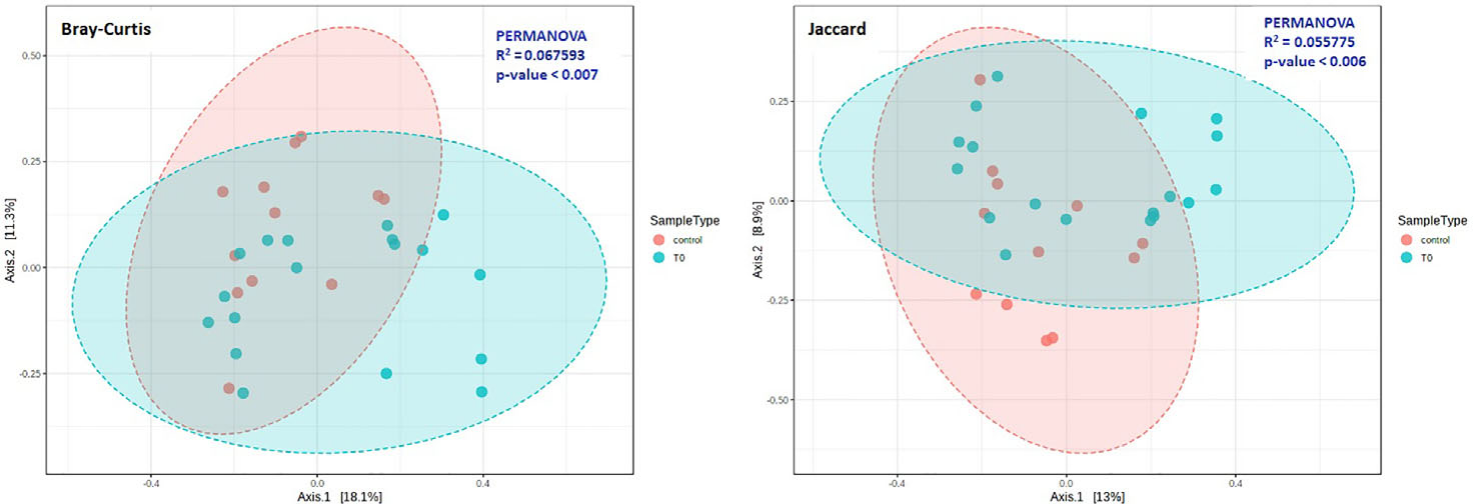
Beta diversity in COVID-19 (T0) and Control groups. Principal coordinate analysis (PCoA) plots were performed by the Bray-Curtis (left) or the Jaccard (right) distance measures. Statistical significance was assessed by PERMANOVA. In both cases we found a significant result (Bray-Curtis: *p* = 0.007; Jaccard: *p* = 0.006), and analysis confirmed that the distance between groups was dependent on the relative abundance of taxa rather than on the type.

The beta diversity was measured by Bray-Curtis (p=0.007) and Jaccard (p=0.006) analysis, *via* the PCoA ordination method, for the evaluation of dissimilarities in patients and controls (**Figure 2**). Our analysis confirmed that the distance between groups was dependent on the relative abundance of taxa rather than on the type (**Figure 2**).

**Figure 3:**
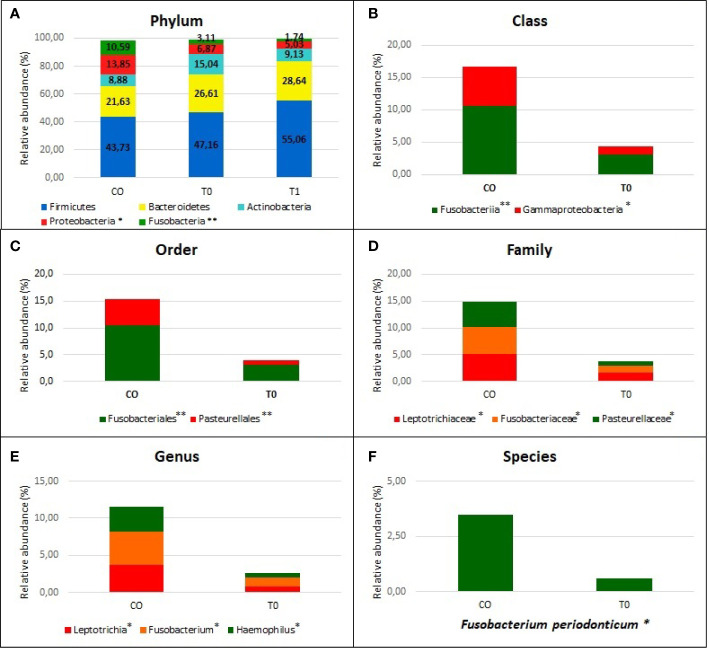
Nasopaheryngeal microbiome composition in COVID-19 patients and Control group. The graphs show the percentage of relative abundance (%) of the all taxonomic levels from Phylum to Species, obtained by using the MicrobAT Suite - SmartSeq. Each column in the plot represents a group, and each color in the column represents the relative abundance (%) for each taxon. In panel **(A)** we show the phyla with average relative abundance greater than 1% in all studied groups; we found two phyla significantly less abundant in COVID-19 patients respect to Controls, Proteobacteria, and Fusobacteria. Not statistically significant difference in taxa abundance was observed when T0 and T1 COVID-19 patients were compared. The other panels **(B–F)** show the taxa abundance from class to up species level significantly different between groups by Kruskal Wallis test. **(B)** class **(C)** order, **(D)** family, **(E)** genus **(F)** species. *p < 0.05; **p < 0.001.

Taxonomic assignment indicated that the nasopharyngeal microbiome in CO and COVID-19 (T0 and T1) individuals consisted of five distinct phyla: Firmicutes (CO = 43.7%, T0 = 49.5%, and T1 = 55.0%), Bacteroidetes (CO = 21.6%, T0 = 24.0%, and T1 = 28.6%), Actinobacteria (CO = 8.9%, T0 = 14.5%, and T1 = 9.1%), Proteobacteria (CO = 13.8%, T0 = 7.4%, and T1 = 5.0%), and Fusobacteria (CO = 10.6%, T0 = 3.2%, and T1 = 1.7%) with a relative abundance >1% in all groups (**Figure 3**).

We found a significant lower abundance of Proteobacteria and Fusobacteria in T0 and T1 with respect to CO (p=0.003 and p<0.0001, respectively). The significant reduction of these phyla among two groups was evaluated from the class up to the genus (p<0.001), by using Kruskal-Wallis test (**Figures 3A–F**). At the genus level, we found a significantly reduced relative abundance of *Leptotrichia, Fusobacterium*, and *Haemophilus* in T0 respect to CO (p= .01, 0.002, and 0.001, respectively) (**Figure 3E**). When the species were considered, the *Fusobacterium periodonticum* (*FP*) resulted as significantly reduced in COVID-19 patients (both at T0 and T1 sampling) as compared to CO subjects (p<0.01) (**Figure 3F**).

No statistical differences were observed when we compared the T0 vs T1: in **Figure 3A** relative abundance of T1 group are reported in relationship to phylum level. In **Supplemental file** the relative abundance (%) of all taxonomic levels from Phylum to Species, obtained by using the MicrobAT Suite – SmartSeq, is reported.

Interestingly, despite the limited number of analyzed patients, we observed a negative correlation between the relative abundance of *Fusobacterium Periodonticum* and the severity of patients’ symptoms (p=0.034, R^2^ = 0.25, R=-0.5).

## Discussion

To date, little is reported about the relationship between microbiome and SARS-CoV-2 infection, particularly due to the difficulties in obtaining peculiar samples in severely symptomatic patients within the emergency departments or intensive care units. So, the standardization of procedures surrounding these types of studies can be biased by preanalytical variables (time of sample drawing, types of patients, personnel operating swabs, etc.) (Kim et al., 2017). Nevertheless, the microbial composition plays a very important role as being an indicator of either healthy or disease status (Wang et al., 2017). Several studies investigated if the microbiota could modify the risk of developing the COVID-19 by evaluating its complications particularly at respiratory level (Bao et al., 2020; De Maio et al., 2020; He et al., 2020; Khatiwada and Subedi, 2020; Shen et al., 2020). The recent evidences reported that some microorganisms are associated with SARS-CoV-2 infection. In particular, Wu et al. found *Leptotrichia buccalis, Veillonella parvula, Capnocytophaga gingivalis, and Prevotella melanogenica* as overexpressed in bronchoalveolar lavage liquid (BALF) of the COVID-19 patients (Wu et al., 2020); Budding et al. reported an association between the composition of the pharyngeal microbiota and SARS-CoV-2 infection, highlighting a less-diverse microbial profile in older individuals. This finding could explain the enhanced susceptibility of the elderly subjects to SARS-CoV-2 infection (Budding et al., 2020).

In our study we found a different microbial composition in nasopharyngeal swabs between COVID-19 patients and controls. Noteworthy, COVID-19 patients showed a significant reduced abundance in Proteobacteria and Fusobacteria as compared to controls; this abundance was confirmed at every taxonomic level of microbiota analysis (from phylum to species). In fact, we found a significant reduction in *Leptotrichia* and *Haemophilus* genus: these findings cannot be considered as superimposable to those reported by Wu et al. since they analyzed broncho-alveolar lavage fluids (BALFs). In this regard, we underline the recovery of BALF is very prone to pre-analytical variability (Pocino et al., 2015) and, therefore, the comparison of data obtained from different research groups is sometimes challenging.

However, by analyzing the microbial content of our patients and controls, we found that *FP* was the most represented in the CO group contrary to the COVID-19 patients. The role of the *FP* is yet unknown and, if it could regulate the ACEs expression, as shown for other intestinal microbes in COVID-19 patients (Geva-Zatorsky et al., 2017), is still to be clarified. Our findings are in agreement with those by Moore et al. who reported that *FP* resulted as significantly decreased three days after SARS-CoV-2 infection (Moore et al., 2020). Our data show that a progressive decline in this species was evident on swabs collected one week after the admission at the hospital department. Interestingly, Yoneda et al. reported that *Fusobacterium periodonticum* is involved in the surface sialylation process (Yoneda et al., 2014). Morniroli et al. reported that some sialic acid residues on the cell surface could work as alternative receptor of the SARS-CoV-2 S protein, in addition to the ACE2, influencing the development of the associated disease. Unfortunately, we cannot currently provide any evidence of a functional relationship (who drives who?) in this regard. Both Mormiroli and Yoneda (Yoneda et al., 2014; Morniroli et al., 2020) have reported that the sialome plays also a defensive role against viral infections, where a reduction of sialic metabolism can reduce the protection of individuals from SARS-CoV-2.

We point out as Fusobacteria exhibit strong adherence to numerous human cell types, probably influencing the modulation of the host inflammatory response: this aspect could explain our findings showing a negative correlation between the relative abundance of *Fusobacterium Periodonticum* and the severity of symptoms. This result could strengthen our hypothesis of a potential protective role of *FP* against SARS-CoV-2. We have planned to deepen this functional aspect in coming experimental settings that, unfortunately, take time, *FP* being a slow growing microorganism.

Our study presents the following strengths: (1) our COVID-19 patients were all enrolled within the same Department; (2) the nasopharyngeal swab collection was always performed using a standardized procedure from the same selected medical staff, to reduce pre-analytical biases; (3) the nasopharyngeal swab collection was performed upon admission for all patients with diagnosis of COVID-19, confirmed by molecular assay, before starting with any drug treatment; (4) the comparison between COVID-19 positive patients and a cohort of healthy controls regarded individuals coming from the same geographical region, reducing the environment-dependent variability; (5) the control group is still negative at SARS-CoV-2 infection, being all the members recruited among healthy people working in our hospital, who are periodically monitored with molecular assay, in compliance with our hospital policy.

Limitations of our study: one of the limitations of this work is represented by small number of patients and controls analyzed. Nevertheless, due to difficulties related to the enrollment of COVID-19 patients, also other similar studies were not able to collect greater cohorts of subjects. Moreover, although our study analyzed a limited number of patients, our patients and controls were all clinically staged by an *ad hoc* medical team at admission and also evaluated in the follow-up, when possible. Further studies are needed to confirm these data on larger patient cohorts. Also, functional *in vitro* studies are ongoing to decipher the molecular mechanisms of interaction between *FP* and SARS-CoV-2.

We also highlight that De Maio et al. (2020) stated that SARS-CoV-2 did not modify the microbiome composition of the patients as compared to the controls: we believe that the lack of any statistical difference reported could be due to the different characteristics of patients enrolled (mainly with mild disease form).

Finally, although the perfect layout for studying microbiota is still challenging to realize, we can consider our findings as the first clearly showing a potential role of Fusobacteria in protecting oral mucosa from SARS-CoV-2 infection. Further research will be performed to define the pathophysiological mechanisms of this apparently protective effect of *Fusobacterium periodonticum* against COVID-19.

## Data Availability Statement

The datasets presented in this study can be found in online repositories. The names of the repository/repositories and accession number(s) can be found below: https://www.ncbi.nlm.nih.gov/sra, SUB8444038.

## Ethics Statement

The studies involving human participants were reviewed and approved by Ethical Committee of the University Federico II of Naples (authorization n.180/20/ES1 on 25.05.2020). The patients/participants provided their written informed consent to participate in this study.

## Author Contributions

CN: execution of experiments, data and statistical analyses, and manuscript writing. IG, BP, and CD: patient recruitment and sample and clinical data collection. MS: technical collaboration to part of experiments. GS: Bioinformatic support. GC: Elaboration and revision of ethical committee forms and documentation. EC: study conception and design, final revision, and editing of the manuscript. All authors have read and agreed to the published version of the manuscript. All authors contributed to the article and approved the submitted version.

## Funding

This work was supported by a grant of Regione Campania, Task Force Covid-19 DGR 140/17 March 2020.

## Conflict of Interest

The authors declare that the research was conducted in the absence of any commercial or financial relationships that could be construed as a potential conflict of interest.
